# The Red Algae Compound 3-Bromo-4,5-dihydroxybenzaldehyde Protects Human Keratinocytes on Oxidative Stress-Related Molecules and Pathways Activated by UVB Irradiation

**DOI:** 10.3390/md15090268

**Published:** 2017-08-25

**Authors:** Mei Jing Piao, Kyoung Ah Kang, Yea Seong Ryu, Kristina Shilnikova, Jeong Eon Park, Yu Jae Hyun, Ao Xuan Zhen, Hee Kyoung Kang, Young Sang Koh, Mee Jung Ahn, Jin Won Hyun

**Affiliations:** 1 Jeju National University School of Medicine and Jeju Research Center for Natural Medicine, Jeju 63243, Korea; meijing0219@hotmail.com (M.J.P.); legna07@naver.com (K.A.K.); rmj5924@naver.com (Y.S.R.); Kristina.shilnikova@gmail.com (K.S.); jeongeon7780@hanmail.net (J.E.P); yujae1113@jejunu.ac.kr (Y.J.H.); zhenaoxuan705@gmail.com (A.X.Z.); pharmkhk@jejunu.ac.kr (H.K.K.); yskoh7@jejunu.ac.kr (Y.S.K.); 2 Laboratory of Veterinary Anatomy, College of Veterinary Medicine, Jeju National University, Jeju 63243, Korea; healthy@jejunu.ac.kr

**Keywords:** 3-bromo-4,5-dihydroxybenzaldehyde, ultraviolet B, matrix metalloproteinase-1, activator protein-1, mitogen-activated protein kinases

## Abstract

Skin exposure to ultraviolet B (UVB) irradiation leads to the generation of reactive oxygen species (ROS). Excessive ROS cause aging of the skin via basement membrane/extracellular matrix degradation by matrix metalloproteinases (MMPs). We recently demonstrated that 3-bromo-4,5-dihydroxybenzaldehyde (BDB), a natural compound of red algae, had a photo-protective effect against UVB-induced oxidative stress in human keratinocytes. The present study focused on the effect of BDB on UVB-irradiated photo-aging in HaCaT keratinocytes and the underlying mechanism. BDB significantly impeded MMP-1 activation and expression, and abrogated the activation of mitogen-activated protein kinases and intracellular Ca^2+^ level in UVB-irradiated HaCaT cells. Moreover, BDB decreased the expression levels of c-Fos and phospho-c-Jun and the binding of activator protein-1 to the *MMP-1* promoter induced by UVB irradiation. These results offer evidence that BDB is potentially useful for the prevention of UVB-irradiated skin damage.

## 1. Introduction

A family of matrix metalloproteinases (MMPs) is secreted and membrane-bound zinc-dependent endopeptidases that have the combined capacity to degrade all of the extracellular matrix components [1]. These enzymes are secreted in response to various stimuli, including ultraviolet (UV), oxidative stress, and cytokines, in various cell types, including keratinocytes and dermal fibroblasts [2]. UVB-induced secretion of MMPs in skin cells is responsible for the degradation of dermal collagen and leads to aging of normal human dermal fibroblasts [3]. UVB is known to induce the expression of MMP-1, -3, and -9 in the normal human epidermis in vivo [4]. Moreover, UVB irradiation stimulates the generation of reactive oxygen species (ROS) and induces the overexpression of MMP-1, -3, and -9 in human fibroblasts, resulting in the destruction of collagen that leads to wrinkle formation and sagging, characteristics of aging skin [5,6,7]. Accordingly, these effects can be reversed by active antioxidant compounds to inhibit the expression of MMPs, including MMP-1, by suppressing mitogen-activated protein kinases (MAPKs) such as extracellular signal-regulated kinase (ERK), c-Jun amino-terminal kinase (JNK), and p38 phosphorylation [8,9]. Activator protein-1 (AP-1) is a nuclear transcription factor complex that acts as a key mediator for UV-induced MMPs and the degradation of extracellular matrix proteins. ROS play central roles in UV-induced MMP expression and secretion including MMP-1 through initiating ERK-mediated AP-1 signaling [10,11]. In addition, increased levels of intracellular Ca^2+^ affect the secretion of MMP-1 induced by UV radiation in keratinocytes [12]. The strategies for preventing this ROS production and consequent signaling cascade can be beneficial in reducing the deleterious effects of UVB rays on the skin. Therefore, the beneficial antioxidant properties of certain natural compounds could be exploited to develop products for effectively protecting the skin from this ROS-associated photo-damage.

3-bromo-4,5-dihydroxybenzaldehyde (BDB) is contained in the red algae *Rhodomela confervoides* [13], *Polysiphonia urceolata* [14], and *Polysiphonia morrowii* [15] that shows free radical scavenging activity [14], anticancer activity [16], and antimicrobial activity [15,17]. Moreover, we recently demonstrated that BDB could protect keratinocytes against UVB-induced damage by removing ROS and directly absorbing UVB rays, thereby reducing injury to the cellular components [18]. To elucidate the underlying mechanisms, the aim of the present study was to elucidate the effects of BDB on MMP-1 activity and expression, intracellular Ca^2+^, and activation of MAPKs and AP-1 in UVB-exposed human HaCaT keratinocytes.

## 2. Results and Discussion

### 2.1. BDB Improves Cell Viability and Scavenges Intracellular ROS Generated in UVB-Irradiated HaCaT Keratinocytes

**Figure 1:**
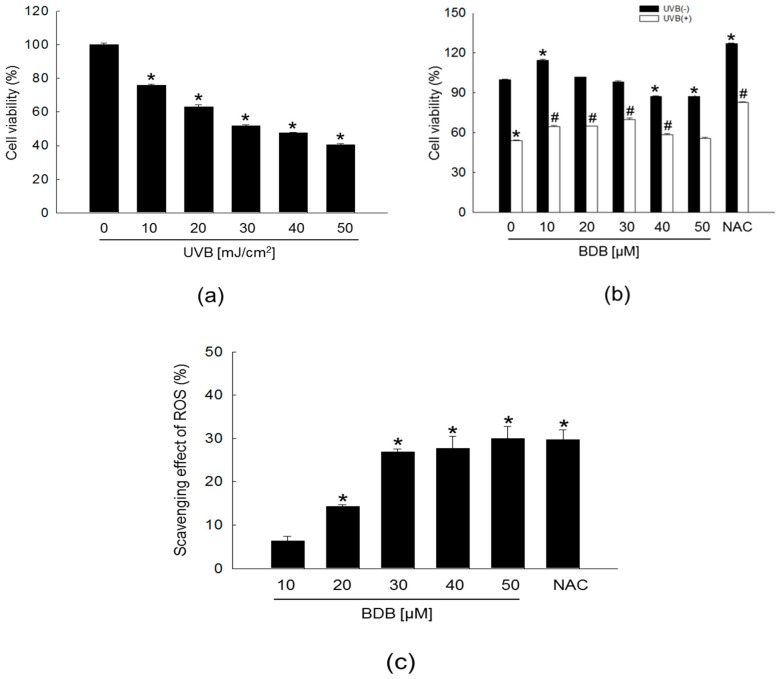
BDB improves cell viability and effectively scavenges intracellular ROS generated in UVB-irradiated HaCaT keratinocytes. (**a**) Cell viability on UVB-irradiated cells at various doses and (**b**) cell viability on various concentrations of BDB in UVB-irradiated cells were performed using the 3-(4,5-dimethylthiazol-2-yl)-2,5-diphenyltetrazolium bromide (MTT) assay. NAC; positive control. (**a**) * *p* < 0.05 vs. the control (*n* = 3 repeats). (**b**) * *p* < 0.05 vs. the control; ^#^ *p* < 0.05 vs. UVB-exposed cells (*n* = 3 repeats). (**c**) Levels of BDB-scavenged intracellular ROS generated by UVB detected spectrofluorometrically by the DCF-DA method. NAC; positive control. Scavenging effects are expressed as percentages. * *p* < 0.05 vs. the control (*n* = 3 repeats).

Light at the UVB wavelength readily penetrates the epidermis and is nearly absorbed in the upper dermis, whereas UVA light penetrates to the deeper layer of the dermis. Whereas, the shorter-wavelength UVB is more effective than UVA in formation of photo-aging [19,20]. We previously demonstrated that BDB showed an absorptive capacity for 280–320 nm wavelengths of UVB rays, which might confer BDB with a photo-protective effect against UVB radiation-induced damage [18]. As a first step toward evaluating these effects and mechanisms, we tested various levels of UVB irradiation to induce sufficient UVB damage to keratinocytes. For effectively studying the cytoprotective effect of BDB, the optimal UVB dose applied to the cells was determined based on the 50% growth inhibitory concentration of 30 mJ/cm^2^ (Figure 1a). BDB was not cytotoxic to human keratinocytes up to 30 μM, but showed significant cytotoxicity at concentrations of 40 μM and 50 μM (Figure 1b, black bars). UVB reduced the cell viability from a control level of ~100% to 54%, and treatment with BDB at 10, 20, and 30 μM significantly increased cell viability to 64, 65, and 70%, respectively (Figure 1b, white bars). These results suggested that BDB has a protective effect on human keratinocytes against UVB radiation. UVB light stimulates the production of ROS to induce oxidative stress [21,22]. In our previous time-course experiment, we found that the maximal ROS levels induced by UVB exposure appeared at 24 h [18]. Therefore, in the present study, intracellular ROS amount formed by UVB radiation were assessed at 24 h by the 2′,7′-dichlorodihydrofluorescein diacetate (DCF-DA). BDB exhibited scavenging activity to intracellular ROS generated by UVB in a concentration-dependent manner, with a steep increase between 20 μM and 30 μM, ultimately reaching an equivalent level (29%) to that observed for 1 mM of N-acetyl cysteine (NAC), a well-documented ROS scavenger that was used as the positive control (Figure 1c). From these results, 30 μM was determined as the optimal concentration of BDB for further study.

### 2.2. BDB Impedes MMP-1 Activity and Expression in UVB-Exposed Keratinocytes

The excessive matrix degradation by UV-induced MMPs observed in various cells, including keratinocytes, fibroblasts, and inflammatory cells, has been suggested to contribute to the debilitation of skin connective tissue [10]. Since UV irradiation-induced ROS production causes skin photo-aging and induces the synthesis of MMPs [5,23], UVB exposure induces a photo-oxidation reaction that disrupts the antioxidant status of the skin and increases cellular ROS levels. Enhanced ROS levels are also accompanied by the activation of ROS-mediated signaling pathways [24,25]. 

**Figure 2:**
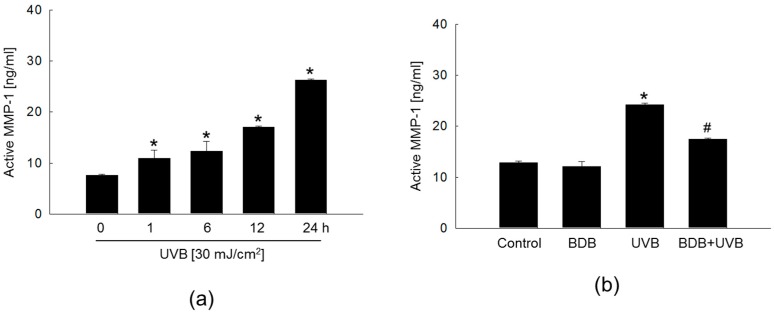
BDB impedes MMP-1 activity in UVB-irradiated HaCaT cells. Cells were irradiated with 30 mJ/cm^2^ of UVB and harvested at the indicated times. (**a**) MMP-1 activity of UVB-irradiated cells at the indicated times and (**b**) MMP-1 activity of BDB-pretreated and UVB-irradiated cells was determined using the human active MMP-1 fluorescent assay kit. (**a**) * *p* < 0.05 vs. 0 h level (*n* = 3 repeats). (**b**) * *p* < 0.05 vs. control; ^#^ *p* < 0.05 vs. UVB-exposed cells (*n* = 3 repeats).

Cells were irradiated to UVB at a dose of 30 mJ/cm^2^, and MMP-1 activity was performed using a fluorescent assay kit. UVB radiation exposure increased the MMP-1 activity in a time-dependent manner, with the highest levels observed at 24 h (Figure 2a). However, this UVB-induced enhancement of MMP-1 activity at 24 h was significantly inhibited by BDB treatment (Figure 2b). Epidermal keratinocytes are the major cellular source of UV-induced MMPs accumulation. The MMPs secreted by the epidermis in response to UV can then diffuse into the dermis where they further accelerate collagen breakdown. Therefore, these results suggest that BDB treatment may effectively inhibit MMP-1 activation and expression in the epidermis, which may manifest as efficient anti-photo-aging effects.

**Figure 3:**
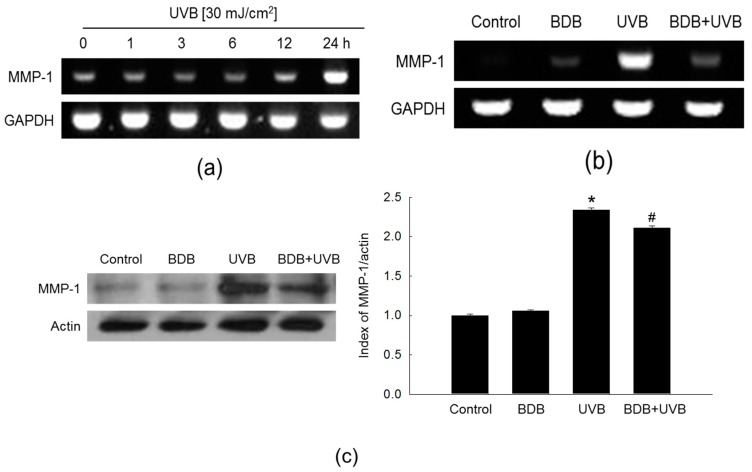
BDB impedes MMP-1 expression in UVB-irradiated HaCaT cells. (**a**) *MMP-1* mRNA expression of UVB-irradiated cells at the indicated times and (**b**) *MMP-1* mRNA expression of BDB-pretreated and UVB-irradiated cells were analyzed by RT-PCR. (**c**) MMP-1 protein expression was analyzed by Western blotting. * *p* < 0.05 vs. control; ^#^ *p* < 0.05 vs. UVB-exposed cells (*n* = 3 repeats). *GAPDH* and actin were used as loading controls in RT-PCR and Western blotting, respectively.

After keratinocytes were exposed to 30 mJ/cm^2^ UVB radiation, reverse transcription-polymerase chain reaction (RT-PCR) analysis showed an increase in the levels of *MMP-1* mRNA in a time-dependent manner, with the highest level exhibited at 24 h (Figure 3a). BDB treatment attenuated the expression of UVB-induced *MMP-1* mRNA and protein at 24 h (Figure 3b,c). 

### 2.3. BDB Impedes MMP-1 Expression through Ca^2+^ Regulation and Suppression of MAPKs Activation in UVB-Exposed HaCaT Keratinocytes

**Figure 4:**
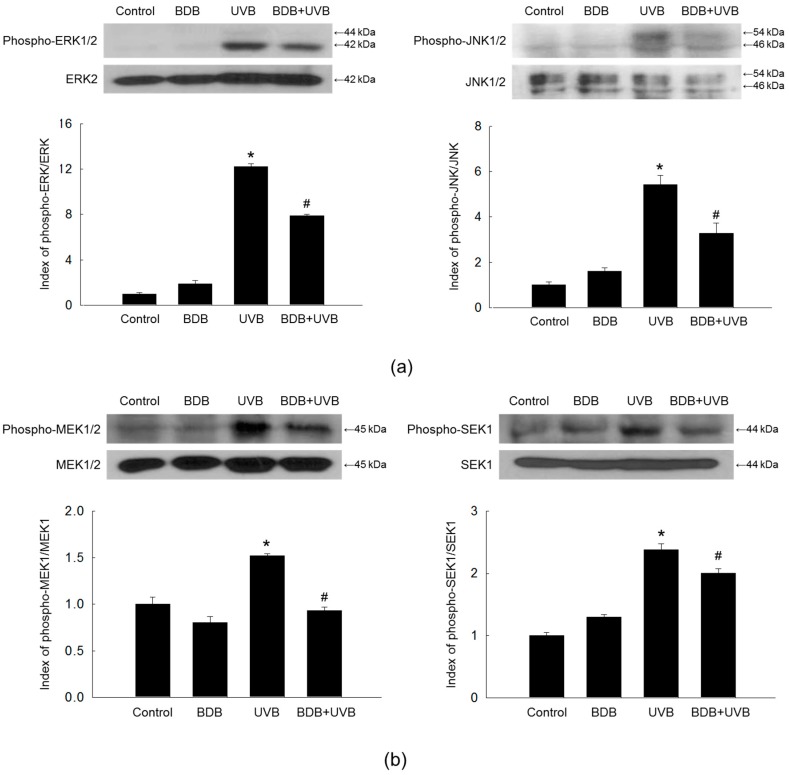
BDB impedes MAPKs activation in UVB-irradiated HaCaT cells. Western blot was performed against (**a**) phospho-ERK1/2, ERK2, phospho-JNK1/2, and JNK1/2 and (**b**) phospho-MEK1/2, MEK1/2, phospho-SEK1, and SEK1. ERK2, JNK1/2, MEK1/2, and SEK1 were used as loading controls, respectively. * *p* < 0.05 vs. control; ^#^ *p* < 0.05 vs. UVB-exposed cells (*n* = 3 repeats).

An increase in the intracellular Ca^2+^ level activates MAPKs [26], which subsequently enhance MMP-1 expression levels on UVB radiation [27]. Western blot analysis showed that UVB light induced the phosphorylation, and activation, of ERK1/2 and JNK1/2 in HaCaT cells, which was suppressed by BDB treatment (Figure 4a). Furthermore, BDB suppressed the UVB-induced active form (phosphorylation) of MAPK kinase (MEK)1/2 and SAPK/ERK kinase (SEK)1, which function upstream of ERK and JNK, respectively (Figure 4b).

**Figure 5:**
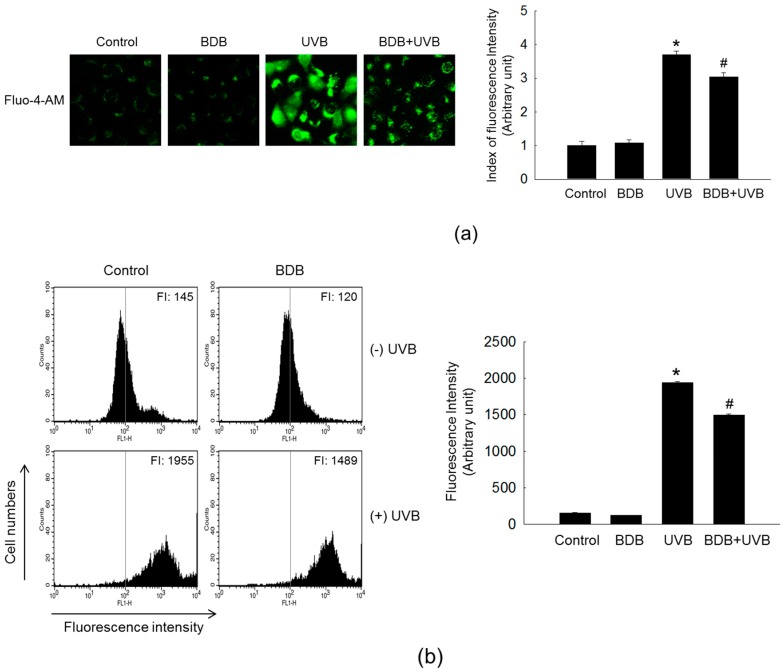
BDB impedes the intracellular Ca^2+^ level in UVB-irradiated HaCaT cells. Ca^2+^ level was detected by (**a**) fluorescence microscopy and (**b**) flow cytometry after staining of Fluo-4-AM. * *p* < 0.05 vs. control; ^#^ *p* < 0.05 vs. UVB-exposed cells (*n* = 3 repeats).

UV irradiation increases the intracellular Ca^2+^ level to regulate the expression level of MMP-1 through the activation of ERK and JNK in HaCaT cells [11,28]. Therefore, the effect of BDB on the intracellular Ca^2+^ level in UVB-irradiated cells was detected by the specific Ca^2+^-fluorescent indicator fluo-4-acetoxymethyl (Fluo-4-AM). Fluorescence microscopy result exhibited a higher intensity of Ca^2+^ fluorescence on UVB exposure compared with control, and the fluorescent intensity was reduced following BDB treatment to UVB-exposed cells (Figure 5a). The confocal microscopy result was confirmed by flow cytometry (Figure 5b).

### 2.4. BDB Impedes AP-1 Expression and Activity in UVB-Exposed HaCaT Keratinocytes

**Figure 6:**
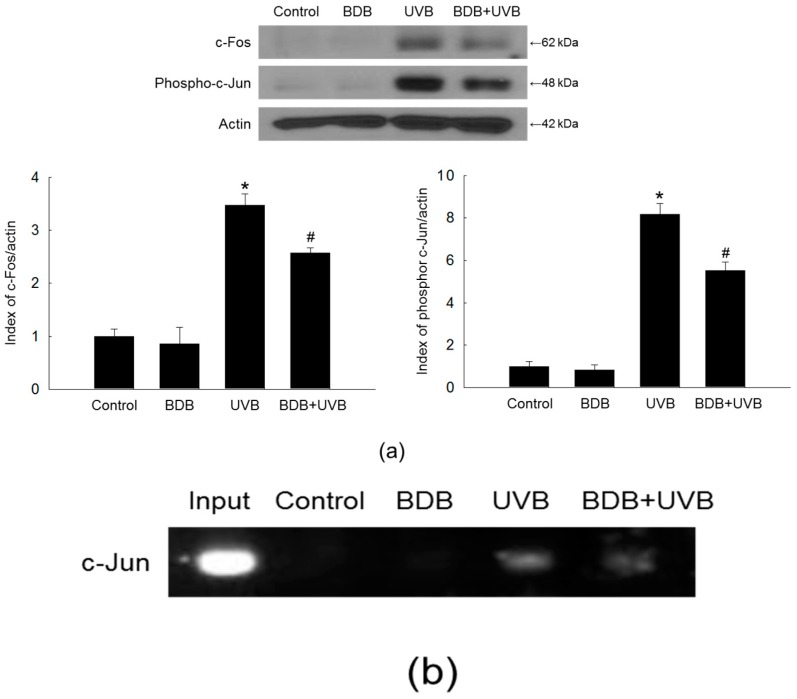
BDB impedes AP-1 expression and activity in UVB-irradiated HaCaT cells. (**a**) Western blot was performed against c-Fos and phospho-c-Jun. Actin was used as a loading control. * *p* < 0.05 vs. control; ^#^ *p* < 0.05 vs. UVB-exposed cells (*n* = 3 repeats). (**b**) AP-1 binding to the *MMP-1* promoter was assessed by ChIP method

The nuclear transcription factor AP-1 consists of either a c-Jun/c-Jun homodimer or a c-Jun/c-Fos heterodimer [29,30], and is a major effector of the MAPK pathways. The *MMP-1* promoter contains a binding site for AP-1; thus, UVB-induced AP-1 activation enhances the MMP-1 expression [31]. Increase of c-Fos leads to the formation of the completely functional heterodimeric AP-1, which then promotes the expression of several MMP members that play major roles in the skin photo-aging [11]. Indeed, UVB treatment markedly stimulated the expression of both c-Fos and phospho-c-Jun in HaCaT cells, whereas BDB treatment suppressed this induction (Figure 6a). BDB attenuated the UVB-induced AP-1 binding to the *MMP-1* promoter, as assessed by a chromatin immunoprecipitation (ChIP) method (Figure 6b).

## 3. Materials and Methods

### 3.1. Cell Culture and UVB Radiation

Human keratinocytes (HaCaT) were obtained from the Amore Pacific Company (Gyeonggi-do, Korea). Cells were maintained at 37 °C in an incubator with a humidified atmosphere of 5% CO_2_. Cells were cultured in RPMI medium 1640 containing 10% heat inactivated fetal bovine serum. Cells were exposed to a dose of 30 mJ/cm^2^ of UVB (CL-1000 M UV Crosslinker, Upland, CA, USA) [32]. BDB was purchased from Matrix Scientific (Columbia, SC, USA) and the purity was above 95%. BDB was dissolved with dimethyl sulfoxide (DMSO) and the concentration of final DMSO in each culture media did not exceed 0.05%.

### 3.2. MTT Method

To provide the optimal condition of UVB radiation, cells were seeded in a 96-well plate at a density of 0.5 × 10^5^ cells/well. Sixteen hours after plating, cells were exposed to 10, 20, 30, 40, and 50 mJ/cm^2^ of UVB. In addition, to assess the effect of BDB (Matrix Scientific, Columbia, SC, USA) against UVB-exposed cells, cells were treated with BDB at 10, 20, 30, 40, and 50 μM and exposed to UVB light at 30 mJ/cm^2^ 1 h later and incubated at 37 °C for one day. An MTT solution (Sigma-Aldrich Co., St. Louis, MO, USA) was added to each well, the formazan crystals were dissolved in DMSO, and cell viability was assessed by measuring the absorbance at 540 nm using a microplate reader [33]. The MTT experiment was repeated three times.

### 3.3. ROS Detection

Cells were seeded in a 96-well plate at a density of 0.5 × 10^5^ cells/well. Sixteen hours after plating, cells were treated with BDB at various concentrations and after 1 h, cells were exposed to UVB light and incubated for 24 h. After the addition of DCF-DA (25 μM) (Sigma-Aldrich Co., St. Louis, MO, USA), the fluorescence of 2′,7′-dichlorofluorescein was detected using a spectrofluorometer [34]. The ROS detection was repeated three times.

### 3.4. MMP-1 Activity

The Fluorokine^®^ E human active MMP-1 fluorescent assay kit (R&D Systems Inc., Minneapolis, MN, USA) was used to assess MMP-1 activity according to the manufacturer’s instructions. Cells were seeded on a 60 mm culture dish at 1 × 10^5^ cells/mL. At 16 h after seeding, cells were treated with BDB (30 μM). After 1 h, the cells were exposed to UVB at a dose of 30 mJ/cm^2^. After 24 h, culture medium was subjected to centrifugation at 1000× *g* for 5 min, and then 150 μL of culture supernatants was mixed with 100 μL of assay diluent buffer in 96-well enzyme-linked immunosorbent assay (ELISA) plates. The plates were shaken for 3 h at room temperature, and then unbound material was washed off. Subsequently, 200 μL of activation reagent (0.5 M APMA in DMSO) was added to each well for pro-MMP-1 activation. The plates were incubated for 2 h at 37 °C in a humidified environment. After washing, 200 μL of fluorogenic substrate was added. After another 20 h at 37 °C, fluorescence was measured using FLUOstar optima microplate reader (BMG Labtech, Cary, NC, USA) with the fluorescent cleavage product was assessed at an excitation wavelength of 320 nm and an emission wavelength of 405 nm. The MMP-1 activity was repeated three times.

### 3.5. RT-PCR

Cells were seeded on a 60 mm culture dish at 1 × 10^5^ cells/mL. At sixteen hours after seeding, cells were treated with BDB (30 μM). After 1 h, the cells were exposed to UVB at a dose of 30 mJ/cm^2^. After 24 h, total RNA was isolated from the cells using easy-BLUE™ total RNA extraction kit (iNtRON Biotechnology, Seoul, Korea). The PCR of *MMP-1* and glyceraldehyde 3-phosphate dehydrogenase (*GAPDH*) were performed as follows: 35 cycles at 94 °C for 20 s, at 55°C for 30 s, and then at 72 °C for 40 s. The primer pairs were designed as follows: human *MMP-1* (Bionics, Seoul, Korea), sense 5′-GGAGGAAATCTTGCTCAT-3′ and antisense 5′-CTCAGAAAGAGCAGCATC-3′; and human *GAPDH* (Bionics)*,* sense 5′-AAGGTCGGAGTCAACGGATTT-3′ and antisense 5′-GCAGTGAGGGTCTCTCTCCT-3′. They were analyzed with image software after gel electrophoresis and staining. 

### 3.6. Western Blot Analysis

Cells were seeded on a 60 mm culture dish at 1 × 10^5^ cells/mL. At 16 h after seeding, cells were treated with BDB (30 μM). After 1 h, the cells were exposed to UVB at a dose of 30 mJ/cm^2^. After 24 h, the cells were harvested and the total protein was isolated from the cells using 100 μL of PRO-PREP^TM^ protein extraction solution (iNtRON Biotechnology). 30 μg of protein was electrophoresed on a 10% sodium dodecyl sulfate-polyacrylamide gel. The electrophoresed proteins were transferred onto membranes, were subsequently incubated with the indicated primary antibodies followed by further the appropriate immunoglobulin G–horseradish peroxidase secondary antibody conjugates. Protein bands were detected using a Western blotting detection system. The antibodies used in these experiments were MMP-1 (Epitomics–an Abcam Company, Burlingame, CA, USA), ERK2 (K-23) (Santa Cruz Biotechnology, Santa Cruz, CA, USA), phospho-ERK1/2 (Cell Signaling Technology, Beverly, MA, USA), JNK1/2 (Cell Signaling Technology), phospho-JNK1/2 (Cell Signaling Technology), MEK1/2 (Cell Signaling Technology), phospho-MEK1/2 (Cell Signaling Technology), SEK1 (Cell Signaling Technology), phospho-SEK1 (Cell Signaling Technology), c-Fos (Cell Signaling Technology), and phospho-c-Jun (Cell Signaling Technology). These primary antibodies were used in 1:1000, secondary antibodies source were rabbits, using goat anti-rabbit IgG (H + L), horseradish peroxidase conjugate, the ratio of 1:10,000. The Western blot analysis was repeated three times.

### 3.7. Detection of Ca^2+^ Level

Cells were seeded in a 4-well chamber slid at a density of 1 × 10^5^ cells/mL. At 16 h after plating, cells were treated with 30 μM BDB, and then exposed to UVB 1 h later. After an additional incubation for 24 h, the cells were loaded with 10 μM Fluo-4-AM (Molecular Probes, Eugene, OR, USA), a Ca^2+^-sensitive fluorescent probe, and incubated for 30 min at 37 °C. This was followed by washing with phosphate-buffered saline to remove any unbound dye. The stained cells were mounted onto a microscope slide with mounting medium (DAKO, Carpinteria, CA, USA). Microscopic images were collected using the FV1200 laser scanning microscopes Olympus FV10-ASW viewer 4.2 (Olympus Corporation, Tokyo, Japan) on a confocal microscope. For flow cytometry, cells were seeded in 6-well plates at a density of 0.8 × 10^5^ cells/well. The cells were then treated under the same conditions. The cells were suspended in PBS containing Fluo-4-AM (10 μM). After incubation at 37 °C for 30 min, analysis was performed. The detection of Ca^2+^ level was repeated three times.

### 3.8. ChIP

The ChIP assay was performed according to the instructions using the SimpleChIP™ enzymatic chromatin IP kit (Cell Signaling Technology). Cells were seeded on a 100 mm culture dish at 1 × 10^5^ cells/mL. At 16 h after seeding, cells were treated with BDB (30 μM). After 1 h, the cells were exposed to UVB at a dose of 30 mJ/cm^2^. After 24 h, the cross-linked chromatin was digested with nuclease according to the instructions, and the c-Jun antibody (Cell Signaling Technology) and the rabbit IgG (Cell Signaling Technology) were added to the chromatin digests. The protein G magnetic beads were added, the immuno-precipitated complexes were eluted with buffer, proteinase K was added and the solution was incubated at 65 °C for 2 h. The immuno-precipitated DNA fragments were purified on spin columns and the recovered DNA was subjected to 35 cycles of PCR. The primers for the *MMP-1* gene promoter (−67 to +94 of the *MMP-1* gene sequence from the transcription starting site, Bionics) were designed as follows: sense 5′-CCTCTTGCTGCTCCAATATC-3′ and antisense 5′-TCTGCTAGGAGTCACCATTTC-3′. The PCR products were separated on agarose gel, DNA bands were stained and analyzed with Image software.

### 3.9. Statistical Analysis

All measurements were made in three independent experiments, and all values are expressed as the mean ± standard error. The results were subjected to analysis of variance, and Tukey’s post hoc test was used to examine differences between conditions. In each case, a *p*-value < 0.05 was considered statistically significant.

## 4. Conclusions

In conclusion, these results suggest that BDB impedes MMP-1 expression by blocking the activity of MAPKs and AP-1 in UVB-irradiated human keratinocytes. BDB, which is primarily derived from red algae, belongs to a large class of naturally occurring phenolic compounds that exhibit antioxidant activity by means of ROS scavenging. Recently, we demonstrated that BDB had a photo-protective effect against UVB-induced oxidative stress in HaCaT cells [18]. In this study, we demonstrated the potential for BDB to protect against skin aging induced by UVB and the underlying molecular mechanism by inhibiting UVB-induced MMP-1 production and blocking the rapid activation of distinct MAPKs (e.g., ERK and JNK) of the MEK–ERK and SEK–JNK pathways in UVB-exposed human HaCaT keratinocytes. Importantly, BDB impeded the UVB-irradiated increase in intracellular Ca^2+^ level, demonstrating its effectiveness in suppressing the UVB-activation of MAPK signal transduction pathways and downstream cellular responses to prevent oxidative damage in HaCaT keratinocytes. These results highlight BDB as a candidate for development of a marine-derived drug to protect against skin photo-aging and diseases.
